# Reproductive performance and sex ratio adjustment of the wild boar (*Sus scrofa*) in South Korea

**DOI:** 10.1038/s41598-022-25626-z

**Published:** 2022-12-16

**Authors:** Seong-Min Lee

**Affiliations:** Korean Mammal Institute, Gunpo, 15823 Republic of Korea

**Keywords:** Evolutionary ecology, Ecology, Zoology

## Abstract

The wild boar (*Sus scrofa*), a polygynous species, rapidly expanded its geographical range and increased its population size in South Korea following the extinction of large carnivores and changes to rural environments. Understanding wild boar reproductive traits and strategies is essential for their effective management; however, studies in this area are lacking. Using samples collected from hunting bags, the relationships between 1) litter size and female weight and 2) fetal sex ratio and female body condition were examined to understand wild boar life-history strategies. Wild boars showed a seasonal breeding pattern that maximized reproduction. Litter size (mean = 5.7 ± 1.7) was correlated with female weight, whereas fetal sex ratio was not explained by female body condition. However, the heaviest ranked fetuses within the litters were male-biased. Wild boars aged three years or less accounted for 90% of the total population, and sexual dimorphism developed from two years of age. Considering that their reproductive strategy is more effective (i.e., early gestation and large litter size) than that of other polygynous species, the Trivers–Willard model was not supported for the wild boars in this study. Instead, females adjusted the sex of the heaviest fetus in the litter to maximize lifetime reproductive success.

## Introduction

The extinction of top predators can lead to an increase in prey populations^1–4^. In South Korea, large carnivores (i.e., Amur tigers [*Panthera tigris altaica*], Amur leopards [*Panthera pardus orientalis*], and Korean grey wolves [*Canis lupus koreanus*]) that could control wild boar (*Sus scrofa* L.) populations were extirpated during the 1900s^5^. Meanwhile, urbanization and changes in rural communities, owing to aging and declining human populations, have contributed to an increase in abandoned farm fields, which provide suitable habitats for wild boars^6, 7^. Consequently, wild boars have rapidly expanded their geographical range across South Korea, and their population size has drastically increased since the early 2000s^8^.

Recent  conflicts between humans and wild boars (i.e., crop damage, presence in downtown areas, and spread of disease) have become a serious concern, increasing the demand for an effective management plan. Although wild boars are the main game species in South Korea^9^, current management strategies are inadequate for regulating population increases due to a lack of understanding about wild boar reproductive traits and life-history strategies.

Wild boars inhabiting urban and agricultural environments in South Korea have an omnivorous diet and substitute natural food resources with crops in the fall in preparation for harsh winters^10^. Several studies conducted in Europe have shown that pulsed resources can affect female reproductive parameters^11–15^; thus, the adaptability of wild boars to different environments may influence their reproductive performance. Wild boars can give birth throughout the year^16–18^ with a mean litter size that varies from three to six depending on the environment (see^18, 19^ for reviews), and sexual maturity in females reached at a body weight of approximately 15–35 kg^15, 20, 21^. Understanding reproductive performance is a vital component for improving future wild boar management strategies (i.e., population modeling, hunting plans, and culling programs). However, no studies have been conducted to elucidate wild boar reproduction in South Korea.

Wild boars have a high reproductive capacity (i.e., large litter size, relatively short gestation period, and early age at first pregnancy) compared with that of similar-sized ungulates^22, 23^. They are also polygynous and sexually dimorphic, with males being larger than females^24, 25^. Trivers and Willard^26^ proposed a model for polygynous and sexually dimorphic species that suggests that mothers in good condition produce more sons, whereas those in poor condition produce more daughters to increase the number of possible progenies, owing to higher reproductive success in females. According to the Trivers–Willard model (TWM), when males have a greater variance in reproductive success than females, mothers maximize their lifetime reproductive success by adjusting their offspring sex ratio in favor of male offspring^27, 28^. Although the TWM has been tested in a variety of vertebrate populations, it remains controversial (see^28, 29^ for reviews). Contradictory results were also obtained in wild boar and domestic pig studies. Studies by Fernández-Llario and Mateos-Quesada^30^ and Meikel et al.^31^ support the TWM in wild boars and domestic pigs, respectively, while those by Servanty et al.^32^ and Mendle et al.^33^ do not. Variation in the sex ratio of progeny is a key variable in understanding the life history strategies and evolutionary fitness of a species^34^. Additionally, to understand lifetime reproductive success, data on the population age structure and sexual dimorphism is essential^27, 35^. Nonetheless, no collection of data on these variables has been conducted in South Korea for wild boars.

Thus, this study aimed to (1) determine the seasonal breeding patterns of wild boars by analyzing the timing of conceptions and births; (2) examine the relationship between the weight of a mother and litter size to understand reproductive traits; (3) identify the age structure and age at which sexual dimorphism of the wild boar population becomes apparent; and (4) test whether the TWM assumptions can be applied to wild boars in South Korea.

## Results

At the two study sites, a total of 253 wild boars (male, *n* = 128; female, *n* = 125) were captured and aged, of which 190 individuals were weighed. Litter size was analyzed from 45 placentae; however, the fetuses of three females could not be sexed and weighed due to being damaged by gunshots.

### Conception and birth

**Figure 1:**
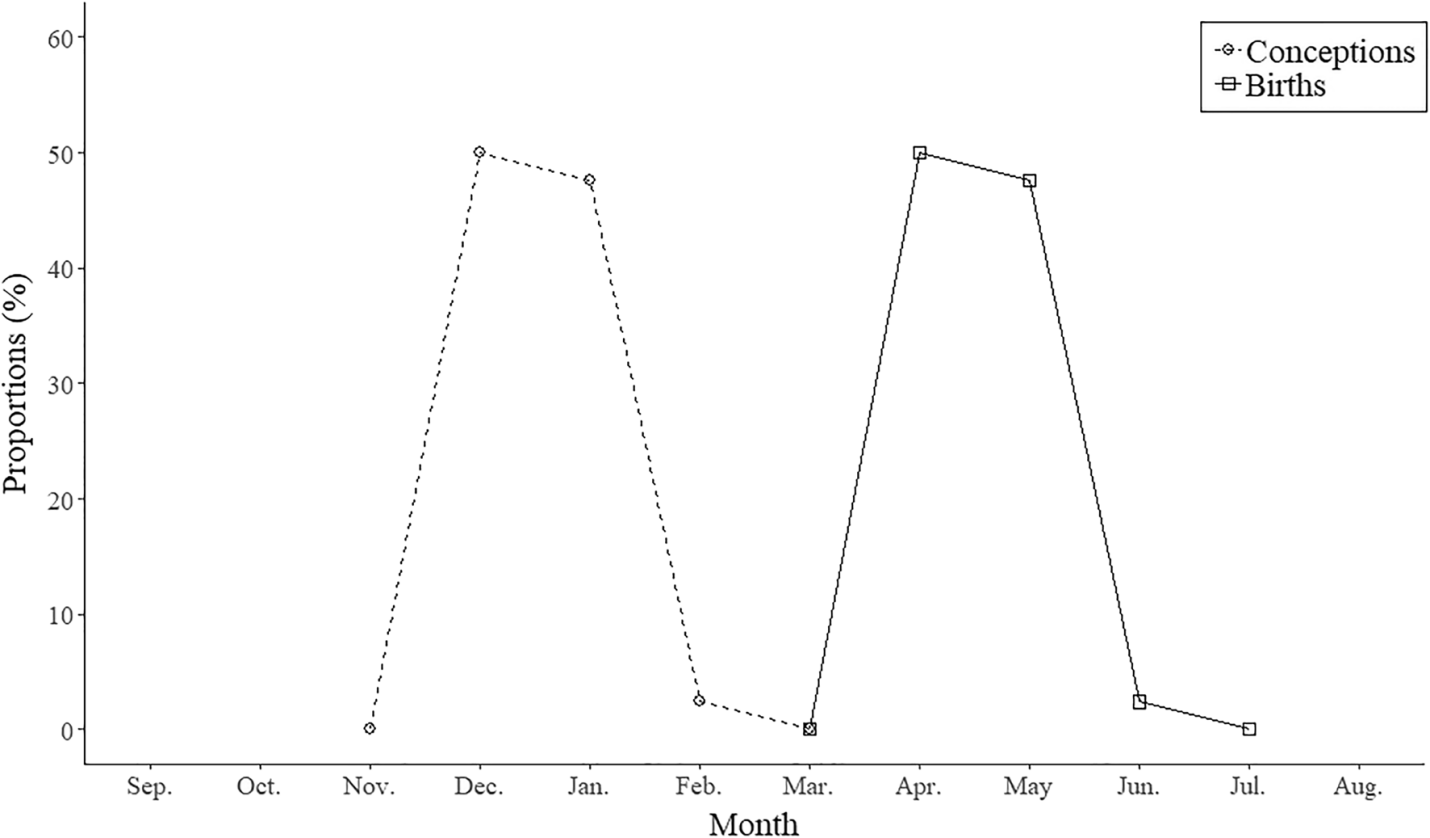
Monthly distributions of conceptions (dashed line with circles) and births (solid line with squares) for wild boars (*n* = 42) collected in South Korea from 2016 to 2018.

The regular mating season for wild boars in this study was from December to February, with the highest number of conceptions in December and January (50% and 45%, respectively), followed by February (2.4%). Births occurred between April and June, the majority of which took place in April and May. The breeding pattern graph was a narrow unimodal type that was concentrated over a specific period, and pregnant females were not found throughout the rest of the year (Fig. 1).

### Litter size

**Figure 2:**
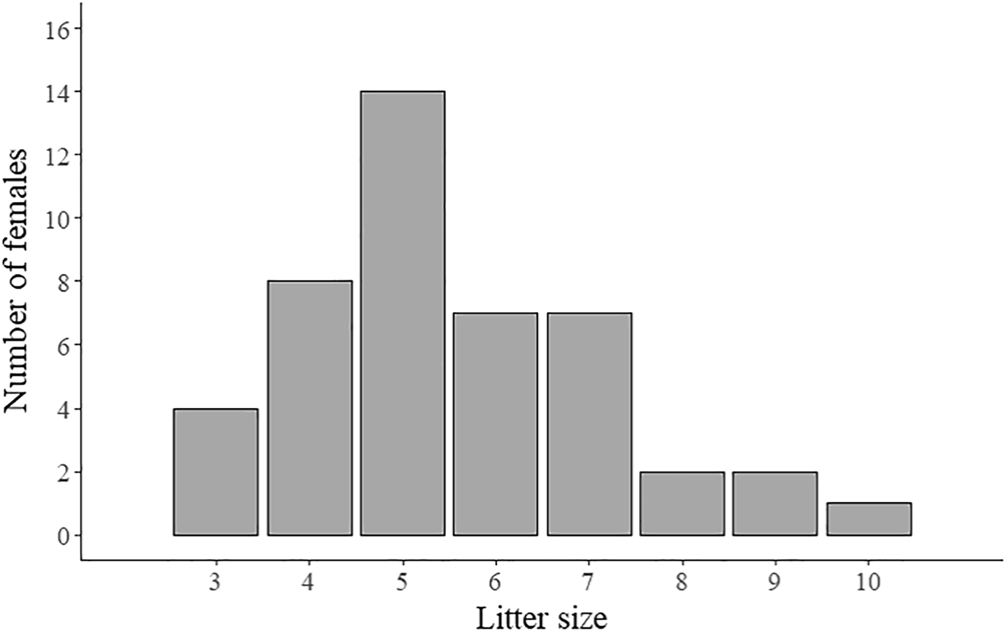
Frequency distribution of wild boar litter size (*n* = 45) collected in South Korea from 2016 to 2018.

**Figure 3:**
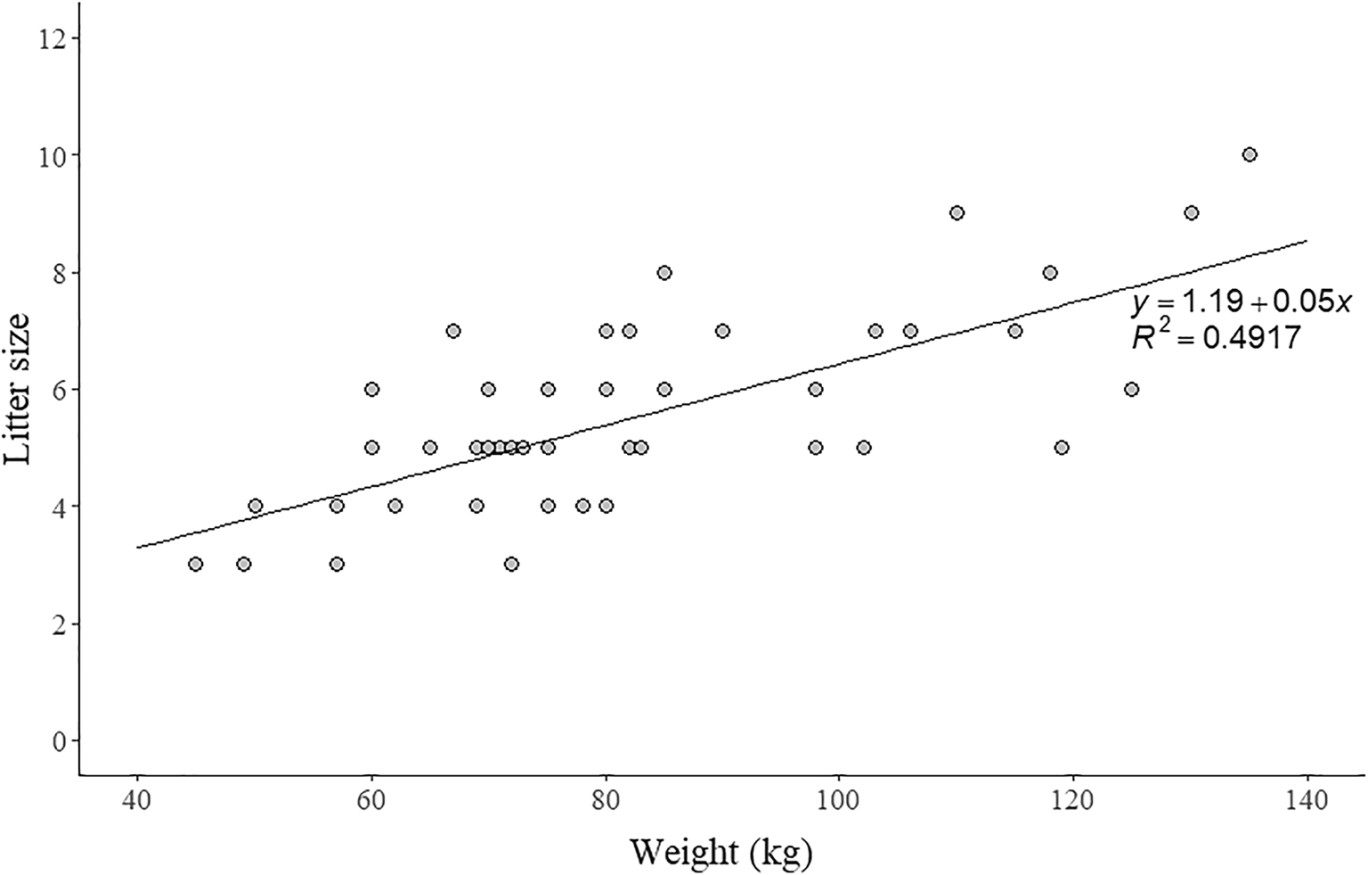
Positive relationship between litter size and female weight (*n* = 45) in a wild boar population in South Korea shown by the linear regression model.

Litter size ranged from three to ten offspring, with five the most common number of offspring (Fig. 2). The mean litter size was 5.7 ± 1.7 SD. The minimum weight and age of the pregnant females were 45 kg and 10 months, respectively. The number of fetuses per litter increased significantly with maternal weight (linear regression; *y* = 1.19 + 0.05*x*, *R*^2^ = 0.4917, *P* < 0.05; Fig. 3).

### Fetal sex ratio

A total of 238 fetuses (male, *n* = 114; female, *n* = 124) were analyzed from 42 pregnant female wild boars. The sex ratio was 0.47 ± 0.18 SD and did not differ significantly from parity for all fetuses (G-test; *G* = 0.4302, df = 1, *P* = 0.5168). The sex ratio of the heaviest ranked fetuses across all litters was male-biased (male, *n* = 28; female, *n* = 14) and significantly differed from unity (sex ratio = 0.67, *G* = 4.7572, *P* = 0.0292); however, that of the second heaviest (male, *n* = 22; female, *n* = 20) and lightest fetuses (male, *n* = 18; female, *n* = 24) were not significantly different (second heaviest: sex ratio = 0.52, *G* = 4.7572, *P* = 0.0292; lightest: sex ratio = 0.42, *G* = 0.8600, *P* = 0.3537).

The average weight and weight rank number of fetuses across all litters by sex were not statistically significant (average weight t-test: t =  − 1.8189, *P* = 0.0702; weight rank number Wilcoxon rank-sum test: *W* = 7634, *P* = 0.1893). Thus, maternal investment was not biased towards a specific sex.

There was no relationship between maternal body condition and fetal sex ratio (weighted logistic regression: *y* =  − 0.064 to 0.0025 × body condition, *Z* =  − 0.738, *P* = 0.461, *n* = 42). Variations in maternal body condition did not account for the offspring sex ratio. Therefore, the results of this study did not support the TWM.

### Population structure

**Figure 4:**
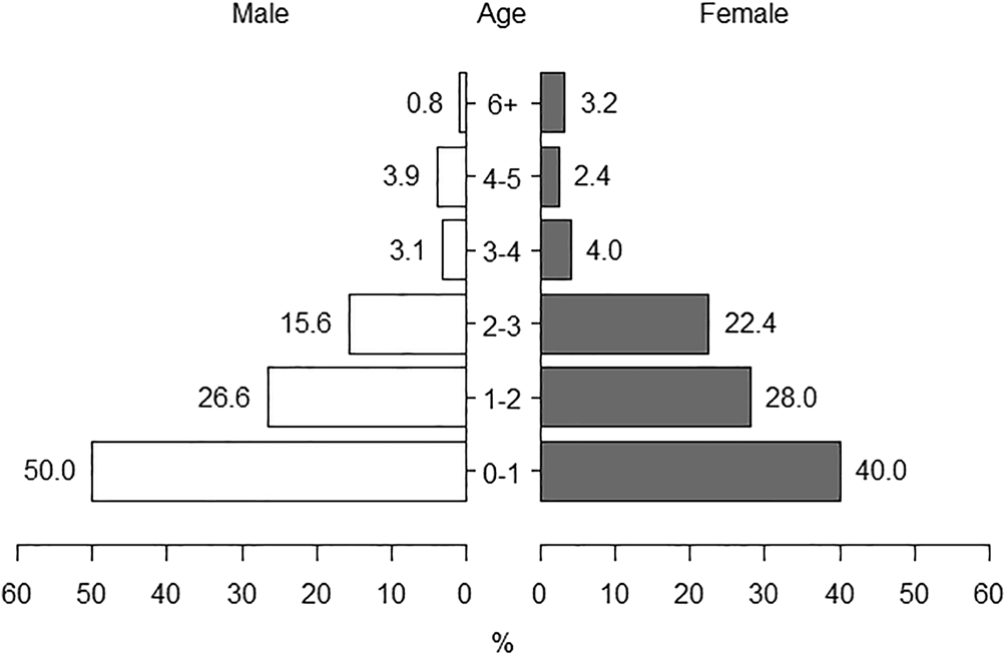
Population age structure of wild boars (*n* = 253) from hunting samples collected in South Korea from 2016 to 2018. Horizontal bars represent the percentage of each age group in both sexes.

One-year-old male and female piglets accounted for 50% and 40% of the wild boar population, respectively. Wild boars aged three years or less accounted for 90% of the total population in both sexes. The proportion of wild boars aged three years or older in the total population decreased considerably (Fig. 4) with a median age of 1.1 years for males and 2.0 years for females.

### Sexual dimorphism

**Figure 5:**
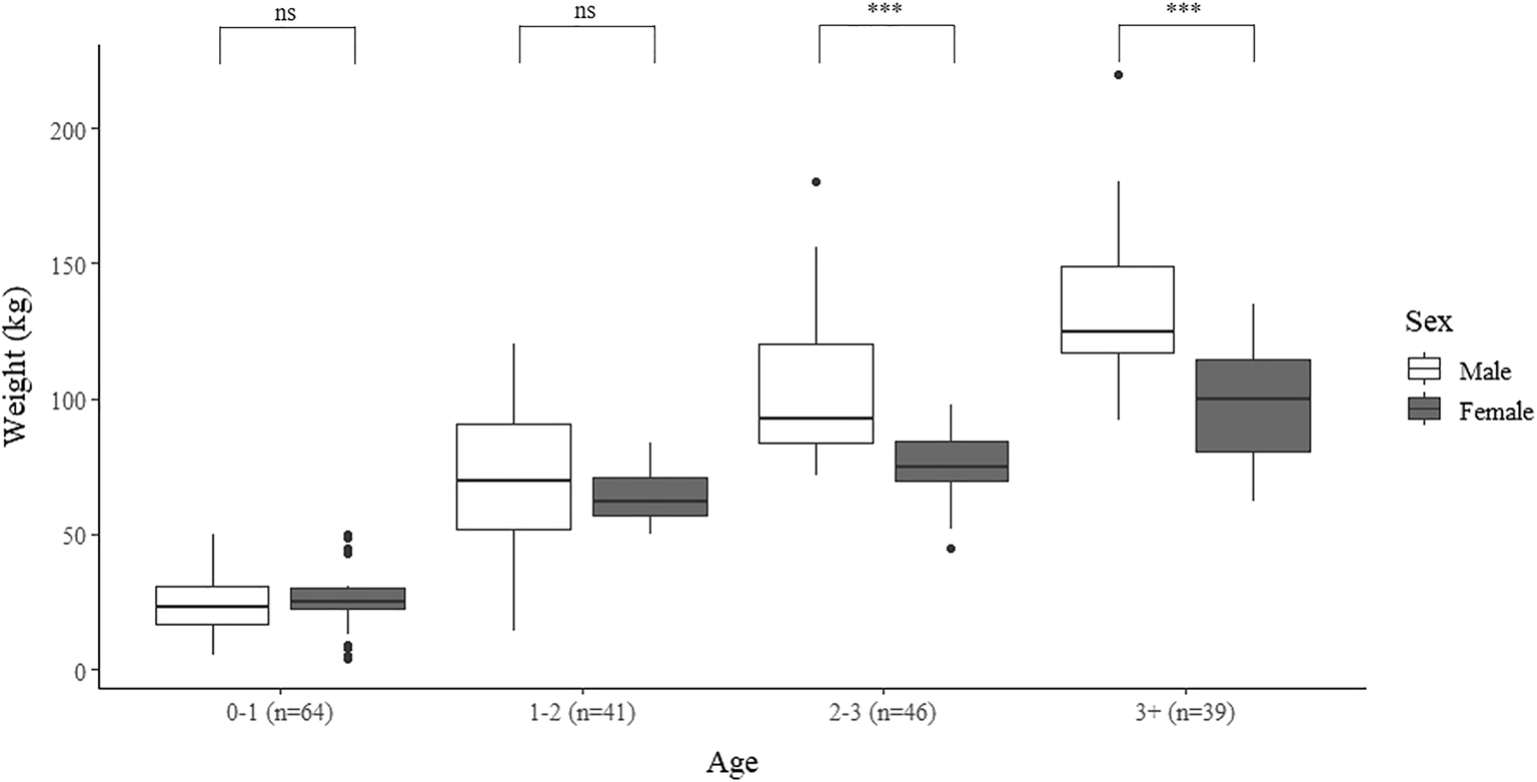
Box-whisker plot of the weight of wild boars (*n* = 190) by sex and age. The horizon line in the box represents the mean weight. Black dots represent outliers. ns = no significant difference; *** *P* < 0.001.

A significant difference was observed in body weight according to sex and age (two-way ANOVA; Sex: *F*_1,188_ = 9.811, *P* < 0.01; Age: *F*_3, 186_ = 183.59, *P* < 0.001). Between sexes, the weight of wild boars under one-year-old and one- to two-years-old did not differ. However, the weight of wild boars over two years of age differed significantly between sexes (Fig. 5).

## Discussion

The reproductive phenology of wild boars is usually a unimodal peak in spring (March–May) or a bimodal peak in spring and summer (Jun–July), and many researchers have reported that wild boars give birth all year-round^16–18, 30, 36^. However, South Korean wild boars in the current study showed a marked seasonal breeding pattern, with the main estrus period in winter and births occurring primarily in spring. These patterns may be representative of food availability, which has shown to affect the breeding seasonality of wild boars^16, 36^. For example, a study conducted in the same area by Lee and Lee^10^ observed that maximum crop consumption of wild boar occurs in the fall. Accordingly, their energy reserves are maximized in early winter, which is consistent with the estrus period of females in this study. Hence, conception in December and January may represent a reproductive strategy to maximize litter size. Based on these findings, focused hunting during the breeding period could serve as an effective management strategy for wild boars in South Korea. The litter size of females was similar to that reported in previous studies (see^18, 19, 22, 37^ for reviews), and the observed positive relationship between female weight and litter size agreed with that reported in European countries^13, 14, 38^.

In the present study, the body condition of mothers did not account for their offspring sex ratio. One of the TWM assumptions is that reproductive success varies more widely among males than females, and sons benefit more from lifetime reproductive success than females. Juvenile male wild boars reach sexual maturity at approximately 6 months of age; however, they can participate in mating competition from approximately two years of age^12, 32^. This is due to their investment in faster growth to compete with adult males for females, which corresponds with the sexual dimorphism results of this study. Although males can monopolize females in a group by outcompeting other males, the proportion of wild boars over three years of age markedly decreased based on the observed age structure of the hunting bags. This demonstrates that the dominance period of the monopolistic male that maximizes its lifetime reproductive success does not last long in high hunting pressure environments. Moreover, when a dominant male is removed by hunting, numerous subordinate males could contribute to the next generation^24^.

In contrast, a female can give birth when it reaches a weight of 27–33 kg, at an age of 6–8 months, or corresponding to 33–41% of their adult weight^15, 20–22^. Consistent with these findings, a ten-month-old juvenile gave birth to a litter of three offspring in current study. In addition, previous studies have found that females can increase their litter size in good environmental circumstances (i.e., suitable rainfall, temperature, and resource availability) and can give birth early under high hunting pressure^14, 15, 19^. These adaptive reproductive traits of female wild boars are effective for increasing reproductive success and are more beneficial for individual fitness compared to those of other ungulates (e.g., bighorn sheep and mountain goats reach sexual maturity at two and four years of age, respectively^15^). Thus, the lifetime reproductive success between male and female wild boars may not be considerably different.

Trivers and Willard^26^ proposed that a high-quality mother produces more sons than daughters to improve individual fitness returns in polygynous species; however, Carranza^39^ reported that producing high-quality males was accompanied by a decrease in litter size. Monopolization by a dominant male is more beneficial than females transferring their genes to progeny if they outcompete others. Therefore, female wild boars may invest their resources in a specific son rather than in a daughter, minimizing the trade-off between producing male offspring and litter size. In this study, the sex ratio of offspring was not influenced by maternal body condition, which corresponds with the results of Servanty et al.^32^ and Fernández-Llario et al.^40^. The fetal sex ratio across litters did not differ from parity; however, the heaviest-ranked offspring were significantly male-biased regardless of female body condition. Similarly, one study by Fernández-Llario et al.^40^ found that 80% of males held the highest positions in the weight ranking within a litter. One explanation could be that the heaviest piglet can gain easier access to a front teat during lactation and monoplize food at both weaning and during early maternal caregiving, in turn, affecting its postnatal growth rate^40–43^. Males with faster growth rates participate in competition for females at an earlier age and are more likely to seize the opportunity for mating when the dominant male is removed. Consequently, female ﻿wild boars in South Korea increase their evolutionary fitness by maximizing litter size and adjusting the sex ratio for only the heaviest fetus, not the whole litter. The placenta sample size in this study was relatively small due to the concentrated breeding pattern in wild boar; thus, further research is needed to examine the relationship between the fetal sex ratio adjustment and female reproductive success.

In conclusion, wild boars inhabiting South Korea had a seasonal breeding pattern of reproduction corresponding to the maximum energy reserve period. Litter size ranged from three to ten and was positively related to female body weight. Three-year-old or younger wild boars constituted 90% of the population, and sexual dimorphism developed from two years of age. The sex ratio of the fetus was not explained by the physical condition of the mother. Considering the more effective reproduction system of wild boars (early reproduction in females and large litter size) compared to that of other polygynous species, this study did not support the TWM. The strategies adopted by female wild boars to increase reproductive success were to maximize their litter size and adjust the sex ratio only in the heaviest fetus. These results suggest that caution should be exercised when testing the TWM in a highly polygynous species.

## Methods

### Study area

Wild boar samples were collected by hunting and culling at two different sites. The first site, Geochang (35° 41′ N, 127° 55′ E; 803.2 km^2^), is located in the southern part of South Korea. This area is characterized by a typical forest-agricultural landscape, and the vegetation is dominated by mixed forests of sawtooth oak (*Quercus acutissima*), Mongolian oak (*Q. mongolica*), and red pine (*Pinus densiflora*). The main crops cultivated are rice (*Oryza* spp.) and apples (*Malus* spp.). The elevation ranges from 200 to 1200 m above sea level, and the mean temperature is 12.0 °C, ranging from − 12.8 to 34.6 °C between winter and summer. The annual precipitation is 1242 mm^44^. The second site, Seoul (37° 41′ N, 127° 11′ E; 605.2 km^2^), is the capital of South Korea and is located in the central part of the country. The landscape of this area is characterized by highly developed cities and fragmented small forests mainly comprising Mongolian oak and red pine. Seoul has climatic conditions very similar to that of Geochang^45^. Hunting is normally conducted from November to February every three years in South Korea, excluding urban areas. Culling is conducted all year-round when wild boars damage crops (agricultural areas) or appear downtown (urban areas). Detailed information about the study area is provided by Lee and Lee^10^.

### Data collection

Samples were collected from 2016 to 2018 by drive hunting using dogs during the day and baiting at night. To normalize population characteristics (i.e., age structure and analysis of sexual dimorphism), samples were collected from two different environments: forest-agricultural in Geochang and urban in Seoul. The placenta samples were collected in Geochang only, due to the regional hunting ban in Seoul and wild boars rarely appear in downtown areas during the main gestation seasons of winter and spring. Drive hunting using dogs is the main method of hunting in South Korea. Dogs drive out wild boars during random encounters, without specific preferences for prey size, sex, or age. For culling programs, local governments reward hunters with 70–80 USD per wild boar capture regardless of its size or sex. The collection of samples using both methods was not biased by the hunters’ preferences or hunting law limitations, and therefore was considered to represent random sampling.

The hunters who participated in hunting and culling programs at both sites held hunting licenses issued by the Korean government and had permission from each local government to conduct annual culling programs. Wild boars were captured within the permitted hunting area or administrative district according to hunting and local culling program guidelines. Hunters reported their results (number of wild boars and capture location) by submitting photos to local government authorities and were subsequently issued a permit for using the wild boar bodies. The author did not participate in hunting or culling activities, and only collected samples and took measurements. As experiments conducted on samples collected from the vertebrate carcasses are not subject to deliberation by the Institutional Animal Care and Use Committee (IACUC), South Korea^46^, this study was performed in accordance with the relevant guidelines and regulations. Wild boars were sexed and weighed to an accuracy of ± 1 kg. Some wild boars captured at night could not be weighed due to the dark environment. The lower jaws were removed to determine the age of all samples, and the placentas were collected from females in gestation. After removing the uterus from the placenta, the number of fetuses was counted for each litter, and every fetus was sexed and weighed to an accuracy of ± 1 g. The age of adult wild boars was determined using tooth eruption and wear methods^47, 48^. The age of the fetuses in days (T) was calculated using the following formula^49^: *T* = (*Ps*^1/3^ + 2.3377)/0.097, where *Ps* is the average fetal fresh weight (g) of the litter. The conception and birth data for each litter were estimated assuming a gestation period of 120 days^22,49,50^. All methods were reported in compliance with ARRIVE guidelines.

### Statistical analysis

A linear regression model was used to determine the relationship between female weight and litter size. To determine the total fetal sex ratio and weight-ranked fetuses, a goodness-of-fit G-test was performed. The observed frequencies were calculated by counting male and female fetuses across litters, and the expected proportion was computed as 0.5 for both sexes. Given that the minimum litter size was three, the sex ratio was tested for the heaviest, second heaviest, and lightest fetuses for all litters.

For each litter, the fetal sex ratio was presented as grouped binary data for binary distribution. The grouped binary data were calculated as the proportion of males (males/[males + females]) within a litter. Weighted logistic regression with a binomial distribution and logit link function was performed to explore the relationship between female body condition and offspring sex ratio. Litter size was set as a weight option in the model to account for the heterogeneity of each sample. Due to the considerable weight variation of wild boars between ages, female body condition was calculated by subtracting the average female weight of each age from the weight of a female. Positive values indicated that a female wild boar weighed more than the female average of the same age, indicating a good body condition, and vice versa for negative values.

To assess the maternal resource investment in offspring sex, fetus weight based on sex was compared across all litters using the two-sample t-test following the Kolmogorov–Smirnov normality test. Thereafter, rank was allocated from first to last in the order of heaviest to lightest weight within the litter. The rank number assigned to each fetus based on sex was also assessed across all litters using the Wilcoxon rank-sum test.

Wild boar weights were square-root transformed for normal distribution. A two-way ANOVA followed by Tukey’s post hoc test for multiple comparisons was conducted to assess the differences between sexes and among ages. Due to the small sample size of wild boars over three years of age, age was categorized into four levels (0–1 y, 1–2 y, 2–3 y, and > 3 y). All statistical analyses were performed using R software version 3.6.0^51^ (Supplementary Information).

## Supplementary Information

Supplementary Information.

## Data Availability

The datasets used and analyzed in this study are available from the corresponding author upon reasonable request.
