# Supplementary material for: Reproductive performance and sex ratio adjustment of the wild boar (Sus scrofa) in South Korea
Source: Sci Rep. 2022 Dec 16;12:21774. doi: 10.1038/s41598-022-25626-z (PMC9758127; doi:10.1038/s41598-022-25626-z)
Supplement: Supplementary file 1 — Supplementary Information. [file 41598_2022_25626_MOESM1_ESM.docx]

**Reproductive performance and sex ratio adjustment of the wild boar (*Sus scrofa*) in South Korea**

Seong-Min Lee

Korean Mammal Institute, Gunpo 15823, Republic of Korea

Supplementary Table S1. Data for 42 females and their fetuses collected in South Korea from 2016 to 2018.

| Female Weight (kg) | Litter Size | Fetal Sex | Fetal Weight (g) |
| --- | --- | --- | --- |
| 57 | 4 | Female | 28 |
|  |  | Male | 36 |
|  |  | Female | 29 |
|  |  | Female | 26 |
| 115 | 7 | Male | 132 |
|  |  | Male | 133 |
|  |  | Female | 124 |
|  |  | Female | 134 |
|  |  | Male | 106 |
|  |  | Female | 113 |
|  |  | Male | 135 |
| 130 | 9 | Female | 91 |
|  |  | Male | 102 |
|  |  | Female | 83 |
|  |  | Female | 87 |
|  |  | Female | 90 |
|  |  | Female | 76 |
|  |  | Female | 97 |
|  |  | Female | 94 |
|  |  | Male | 86 |
| 135 | 10 | Male | 256 |
|  |  | Male | 235 |
|  |  | Female | 294 |
|  |  | Male | 254 |
|  |  | Male | 262 |
|  |  | Male | 243 |
|  |  | Female | 290 |
|  |  | Male | 227 |
|  |  | Female | 129 |
|  |  | Male | 311 |
| 82 | 7 | Male | 49 |
|  |  | Female | 59 |
|  |  | Female | 59 |
|  |  | Female | 61 |
|  |  | Male | 68 |
|  |  | Female | 56 |
|  |  | Male | 60 |
| 125 | 6 | Male | 572 |
|  |  | Male | 442 |
|  |  | Female | 612 |
|  |  | Female | 585 |
|  |  | Female | 547 |
|  |  | Female | 526 |
| 82 | 5 | Male | 162 |
|  |  | Female | 208 |
|  |  | Female | 229 |
|  |  | Male | 194 |
|  |  | Female | 163 |
| 90 | 7 | Male | 549 |
|  |  | Female | 542 |
|  |  | Male | 595 |
|  |  | Male | 597 |
|  |  | Female | 549 |
|  |  | Male | 642 |
|  |  | Male | 597 |
| 118 | 8 | Male | 205 |
|  |  | Female | 191 |
|  |  | Male | 196 |
|  |  | Female | 151 |
|  |  | Male | 211 |
|  |  | Female | 197 |
|  |  | Female | 196 |
|  |  | Female | 188 |
| 83 | 5 | Male | 27 |
|  |  | Male | 31 |
|  |  | Female | 31 |
|  |  | Male | 37 |
|  |  | Male | 37 |
| 69 | 5 | Female | 51 |
|  |  | Female | 61 |
|  |  | Female | 56 |
|  |  | Male | 51 |
|  |  | Female | 63 |
| 80 | 7 | Female | 775 |
|  |  | Female | 736 |
|  |  | Male | 748 |
|  |  | Male | 778 |
|  |  | Male | 725 |
|  |  | Male | 844 |
|  |  | Female | 599 |
| 67 | 7 | Male | 77 |
|  |  | Male | 72 |
|  |  | Male | 80 |
|  |  | Female | 77 |
|  |  | Female | 61 |
|  |  | Male | 79 |
|  |  | Male | 76 |
| 119 | 5 | Female | 139 |
|  |  | Male | 149 |
|  |  | Female | 142 |
|  |  | Male | 132 |
| 102 | 5 | Male | 420 |
|  |  | Male | 387 |
|  |  | Female | 405 |
|  |  | Female | 408 |
|  |  | Male | 424 |
| 57 | 3 | Male | 336 |
|  |  | Female | 333 |
|  |  | Female | 323 |
| 60 | 6 | Female | 240 |
|  |  | Female | 261 |
|  |  | Female | 269 |
|  |  | Female | 251 |
|  |  | Female | 264 |
|  |  | Male | 276 |
| 75 | 6 | Male | 484 |
|  |  | Female | 468 |
|  |  | Male | 466 |
|  |  | Female | 480 |
|  |  | Female | 312 |
|  |  | Male | 449 |
| 75 | 4 | Male | 260 |
|  |  | Female | 276 |
|  |  | Female | 277 |
|  |  | Male | 281 |
| 71 | 5 | Female | 281 |
|  |  | Female | 303 |
|  |  | Male | 304 |
|  |  | Male | 271 |
|  |  | Female | 314 |
| 72 | 3 | Male | 192 |
|  |  | Female | 178 |
|  |  | Male | 188 |
| 65 | 5 | Male | 491 |
|  |  | Female | 465 |
|  |  | Male | 469 |
|  |  | Male | 384 |
|  |  | Female | 451 |
| 72 | 5 | Male | 117 |
|  |  | Male | 117 |
|  |  | Female | 119 |
|  |  | Male | 110 |
|  |  | Female | 114 |
| 98 | 5 | Male | 294 |
|  |  | Male | 296 |
|  |  | Female | 290 |
|  |  | Female | 281 |
|  |  | Male | 317 |
| 73 | 5 | Male | 192 |
|  |  | Male | 184 |
|  |  | Male | 201 |
|  |  | Female | 186 |
|  |  | Female | 191 |
| 78 | 4 | Female | 734 |
|  |  | Female | 692 |
|  |  | Male | 773 |
|  |  | Male | 757 |
| 62 | 4 | Female | 560 |
|  |  | Female | 515 |
|  |  | Female | 471 |
|  |  | Male | 518 |
| 69 | 4 | Female | 150 |
|  |  | Female | 100 |
|  |  | Male | 176 |
|  |  | Male | 147 |
| 102 | 5 | Male | 35 |
|  |  | Male | 52 |
|  |  | Male | 37 |
|  |  | Female | 39 |
|  |  | Female | 38 |
| 75 | 5 | Female | 8 |
|  |  | Female | 7 |
|  |  | Female | 9 |
|  |  | Female | 8 |
|  |  | Male | 7 |
| 70 | 6 | Female | 133 |
|  |  | Male | 125 |
|  |  | Male | 108 |
|  |  | Female | 144 |
|  |  | Male | 148 |
|  |  | Male | 131 |
| 70 | 5 | Male | 528 |
|  |  | Male | 528 |
|  |  | Male | 530 |
|  |  | Male | 543 |
|  |  | Female | 551 |
| 106 | 7 | Female | 22 |
|  |  | Female | 24 |
|  |  | Male | 21 |
|  |  | Female | 23 |
|  |  | Female | 18 |
|  |  | Female | 19 |
|  |  | Male | 22 |
| 103 | 7 | Female | 16 |
|  |  | Female | 21 |
|  |  | Female | 22 |
|  |  | Male | 16 |
|  |  | Male | 19 |
|  |  | Male | 21 |
|  |  | Male | 20 |
| 85 | 6 | Female | 77 |
|  |  | Female | 72 |
|  |  | Female | 72 |
|  |  | Female | 64 |
|  |  | Male | 79 |
|  |  | Male | 79 |
| 80 | 4 | Female | 47 |
|  |  | Female | 46 |
|  |  | Female | 52 |
|  |  | Male | 54 |
| 85 | 8 | Female | 29 |
|  |  | Female | 34 |
|  |  | Female | 32 |
|  |  | Male | 27 |
|  |  | Female | 34 |
|  |  | Female | 32 |
|  |  | Female | 31 |
|  |  | Male | 29 |
| 98 | 6 | Female | 497 |
|  |  | Male | 508 |
|  |  | Male | 523 |
|  |  | Female | 530 |
|  |  | Female | 452 |
|  |  | Female | 520 |
| 60 | 5 | Female | 532 |
|  |  | Male | 593 |
|  |  | Female | 530 |
|  |  | Male | 628 |
|  |  | Female | 567 |
| 80 | 6 | Male | 275 |
|  |  | Male | 243 |
|  |  | Male | 171 |
|  |  | Female | 313 |
|  |  | Male | 275 |
|  |  | Female | 166 |
| 45 | 3 | Male | 40 |
|  |  | Female | 35 |
|  |  | Female | 27 |
| 110 | 9 | Female | 741 |
|  |  | Male | 880 |
|  |  | Male | 748 |
|  |  | Female | 779 |
|  |  | Male | 668 |
|  |  | Male | 684 |
|  |  | Female | 756 |
|  |  | Male | 686 |
|  |  | Female | 802 |
